# Tetraspanin SfCD9 as a Key Membrane Binding Factor of SRBSDV P10 Facilitates Viral Entry Into *Sogatella furcifera* Midgut Epithelial Cells via Clathrin‐Mediated Endocytosis

**DOI:** 10.1111/mpp.70177

**Published:** 2025-11-16

**Authors:** Shibo Gao, Liyan Li, Ming Zeng, Li Xie, Jingjing Li, Xueping Zhou, Jianxiang Wu

**Affiliations:** ^1^ State Key Laboratory of Rice Biology and Breeding, Zhejiang Key Laboratory of Biology and Ecological Regulation of Crop Pathogens and Insects, Institute of Biotechnology Zhejiang University Zhejiang Hangzhou People's Republic of China; ^2^ Hainan Institute of Zhejiang University Sanya People's Republic of China; ^3^ Analysis Center of Agrobiology and Environmental Sciences Zhejiang University Hangzhou China; ^4^ State Key Laboratory of Rice Biology and Breeding, and Zhejiang Provincial Key Laboratory of Crop Germplasm, The Advanced Seed Institute Zhejiang University Hangzhou China; ^5^ State Key Laboratory for Biology of Plant Diseases and Insect Pests, Institute of Plant Protection Chinese Academy of Agricultural Sciences Beijing People's Republic of China

**Keywords:** clathrin‐mediated endocytosis, membrane binding factor, SfCD9, *Sogatella furcifera*, southern rice black‐streaked dwarf virus (SRBSDV), SRBSDV P10

## Abstract

Southern rice black‐streaked dwarf virus (SRBSDV), transmitted by *Sogatella furcifera* , causes significant rice yield losses in Asia. So far, the mechanism by which SRBSDV traverses the midgut barrier to establish infection in *S. furcifera* midgut epithelial cells remains unknown. Here, we show that SRBSDV P10, the major outer capsid protein, enters *S. furcifera* midgut epithelial and haemolymph cells through interacting with the tetraspanin SfCD9 highly expressed in midgut and haemolymph cells. SfCD9 co‐localises with SRBSDV P10 and relocates it from the endoplasmic reticulum (ER) to the cytomembrane of co‐expressing Sf9 cells. SfCD9 localises on the cell membrane and in the cytoplasm in nonviruliferous *S. furcifera* midgut epithelial cells. SRBSDV P10 and SfCD9 colocalised on the midgut epithelial cell membrane of viruliferous *S. furcifera* at 2 days post‐virus feeding (dpvf), and predominantly colocalised in epithelial cell cytoplasm at 6 dpvf. Knockdown of *SfCD9* or oral delivery of the anti‐SfCD9 antibody significantly inhibited SRBSDV invasion in *S. furcifera* . SRBSDV from infected rice crude extracts can enter SfCD9‐expressing Sf9 cells, but not wild‐type Sf9 cells. SfCD9 serves as a key membrane binding factor for SRBSDV entry into vector midgut epithelial cells via clathrin‐mediated endocytosis. Collectively, these findings offer valuable insights into SRBSDV transmission and identify SfCD9 as a potential target to disrupt viral transmission.

## Graphical abstract

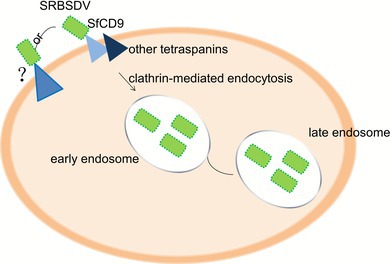

## 1 Introduction

Approximately 30% of the global crop production is lost each year due to virus diseases (Savary et al. 2019). Among these plant virus diseases, the insect‐transmitted virus diseases are the major threat to global rice production, particularly the production in Asia where rice serves as the staple food for more than half of the world's population (Jones 2021).

To date, about 76% of the plant viruses are known to be transmitted by insect vectors in fields (Hogenhout et al. 2008). For arboviruses, such as southern rice black‐streaked dwarf virus (SRBSDV), they need to overcome multiple physiological and immunological barriers in their vectors for successful transmission in fields (Wei and Li 2016). Arbovirus transmission begins with virus acquisition by arthropods through feeding on infected plants followed by virions' entry into the midgut epithelium, the first critical barrier in the infection process. Once in the midgut tissues, the viruses must evade both cellular immunity (Cardoso‐Jaime et al. 2022) and humoral immunity (Monteiro et al. 2018), and subsequently breach the haemolymph barrier when arbovirus in midgut epithelial cells enters the haemolymph. Through haemolymph circulation, the virus can reach the salivary glands and break the salivary gland barrier. Finally, arbovirus in saliva is transmitted to new host plants through feeding on them (Whitfield et al. 2015). To complete this multistepped process, specific interactions between viral and insect proteins are essential for virus entry into insect cells and transmission competence (Dietzgen et al. 2016).

Viruses typically bind to one or more receptors to overcome the cell membrane barrier to invade host cells (Maginnis 2018). Multiple viral receptors have been reported in insects, including sialylated glycans, cell adhesion molecules (e.g., immunoglobulin superfamily members and integrins) and tetraspanins (Grove and Marsh 2011). Tetraspanins, a protein family characterised by conserved four‐transmembrane domains, organise specialised membrane microdomains known as tetraspanin‐enriched domains that serve as platforms for the clustering of receptors, signalling molecules and adhesion proteins (Stipp et al. 2003). Tetraspanins are also known to regulate multiple cellular biological processes, such as cell–cell fusion and endocytosis, which are frequently exploited by various pathogens (Li et al. 2025; Malla and Kamal 2022; Masciopinto et al. 2001; Wuestenhagen et al. 2016). Despite the prominent roles of tetraspanins in mediating human and animal virus invasions, their roles in insect transmission of plant viruses are largely unexplored.

SRBSDV belongs to the genus *Fijivirus*, in the family *Reoviridae*, and was first reported in southern China in 2001. SRBSDV often causes severe damage to rice production in Asian countries (Zhang et al. 2008; Zhou et al. 2008; Wu et al. 2024). Typical disease symptoms on the SRBSDV‐infected rice plants are plant dwarfism, dark green leaves and streaked, white or black waxy galls on stems (Zhou et al. 2013). Because panicles of the SRBSDV‐infected rice plants develop poorly, the rice yield of the infected plants is very low (Zhou et al. 2013). SRBSDV is transmitted by white‐backed planthopper ( *Sogatella furcifera* ) in a persistent, circulative‐propagative manner (Jia et al. 2012; Liu et al. 2010). The SRBSDV genome consists of 10 double‐stranded RNA segments (S1–S10) that encode 13 proteins (Wang et al. 2010). Of these proteins, the segment S10‐encoded P10 protein is the major outer capsid protein. The nonstructural protein P7‐1 can form tubular or fibrillar structures to facilitate virus diffusion in *S. furcifera* (Jia et al. 2014). Recently, Zhang and colleagues reported that SRBSDV hijacks autophagosomes in its vector to prevent their fusion with lysosomes, which promotes viral infection (Zhang et al. 2023). We speculated that SRBSDV might use its major outer capsid protein P10 to bind to *S. furcifera* cell surface prior to its entry into vector cells. Elucidation of the molecular mechanism regulating the interaction between the insect key binding factors and SRBSDV P10 could help us to better understand the transmission of this virus in the field.

In this work, we fed a green fluorescent protein (GFP)‐tagged SRBSDV P10 (P10‐GFP) to *S. furcifera* and found through confocal microscopy that this fusion can enter *S. furcifera* midgut epithelial cells and then the haemolymph. We then performed split‐ubiquitin yeast two‐hybrid assays, using SRBSDV P10 as the bait, to screen a *S. furcifera* cDNA library. Through this screen assay, we identified *S. furcifera* CD9 (SfCD9), a tetraspanin homologue known to play key roles in membrane fusion and protein trafficking, that interacted with SRBSDV P10. Subsequent pull‐down and co‐immunoprecipitation assays confirmed the in vivo and in vitro interaction between P10 and SfCD9. Immunolabelling assay results showed that SRBSDV P10 colocalised with SfCD9 on the apical membrane of midgut epithelial cells. Silencing of *SfCD9* expression through microinjecting dsSfCD9 into *S. furcifera* significantly reduced SRBSDV accumulation in *S. furcifera* , and feeding *S. furcifera* with an anti‐SfCD9 antibody prevented SRBSDV from infecting *S. furcifera* . Furthermore, we have found that SRBSDV could enter SfCD9‐expressing *Spodoptera frugiperda* 9 (Sf9) cells directly and SRBSDV could invade *S. furcifera* midgut epithelial cells via clathrin‐mediated endocytosis. Collectively, we conclude that SRBSDV can utilise the *S. furcifera* tetraspanin system, through interaction with SfCD9, to invade midgut cells, and SfCD9 is a potential target to block SRBSDV transmission.

## 2 Results

### 2.1 SRBSDV P10 Serves as an Insect Receptor Recognition Protein and Mediates SRBSDV Entry Into *S. furcifera*

**FIGURE 1:**
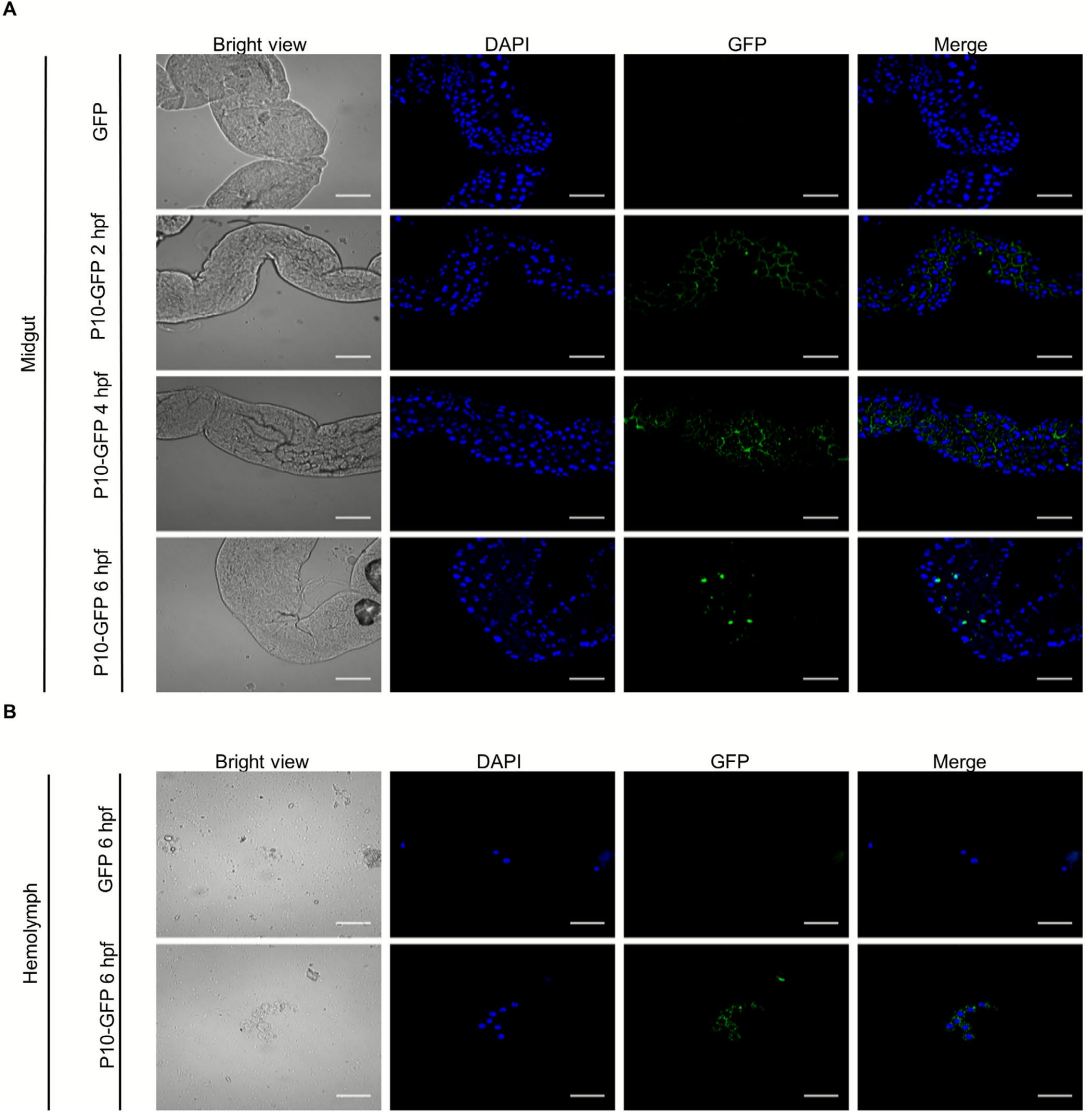
Southern rice black‐streaked dwarf virus (SRBSDV) P10‐GFP fusion protein can enter *Sogatella furcifera* midgut epithelial and haemolymph cells. (A) *S. furcifera* was fed with the P10‐GFP fusion or GFP alone. At 2 h post‐feeding (hpf), green fluorescence in the midgut epithelial cells from the P10‐GFP‐fed *S. furcifera* was observed through confocal microscopy, and found that the P10‐GFP fusion (green) was located mainly on the surface of midgut epithelial cell membranes. At 4 hpf, the P10‐GFP fusion protein was found to form punctae on the epithelial cell membrane and in cytoplasm. At 6 hpf, most of the P10‐GFP fusion protein was found in the cytoplasm, and a small fraction of P10‐GFP fusion protein was still found on the epithelial cell membrane. Scale bar, 50 μm. (B) At 6 hpf, the P10‐GFP fusion protein was also observed in the cytoplasm of haemolymph cells. DAPI staining signal indicates nuclei (blue). In this experiment, no green fluorescence was observed in the haemolymph cells of the GFP‐fed *S. furcifera* . Scale bar, 20 μm.

Plant virus capsid proteins are crucial for virus invasion and intercellular and systemic movement in their vectors. In this study, we investigated the role of SRBSDV P10 during virus invasion in its insect vector. We first expressed SRBSDV P10‐GFP fusion or GFP in *S. frugiperda* 9 (Sf9) cells using a baculovirus expression system. After purification, the SRBSDV P10‐GFP fusion or GFP was used to feed *S. furcifera* followed by confocal microscopy observation. At 2 h post‐feeding (hpf), SRBSDV P10‐GFP was mainly observed on the midgut cell membrane surface. At 4 hpf, the fusion protein was found on the midgut cell membrane and in the cytoplasm with punctate distribution. At 6 hpf, the P10‐GFP appeared mainly in the midgut cell cytoplasm as perinuclear punctae and also appeared in the haemolymph cell cytoplasm (Figure 1A,B). In this experiment, GFP alone was not observed in the midgut cells and haemolymph cells of the GFP‐fed *S. furcifera* control (Figure 1A,B), indicating that SRBSDV P10 is crucial for the entry of SRBSDV into *S. furcifera* midgut epithelial cells.

### 2.2 SRBSDV P10 Interacts With SfCD9 In Vitro and In Vivo

To identify which *S. furcifera* protein(s) could interact with SRBSDV P10, we conducted split‐ubiquitin yeast two‐hybrid (SU‐Y2H) assays using the SRBSDV P10 as the bait to screen a *S. furcifera* cDNA library. Based on this screening, a total of 46 positive clones were identified and sequenced. After removing the duplicated gene segments, a total of 21 unique genes were obtained. These 21 unique genes were annotated through BLASTX searching the GenBank database (Table S1). One of the annotated genes has been reported to encode a tetraspanin family protein SfCD9.

**FIGURE 2:**
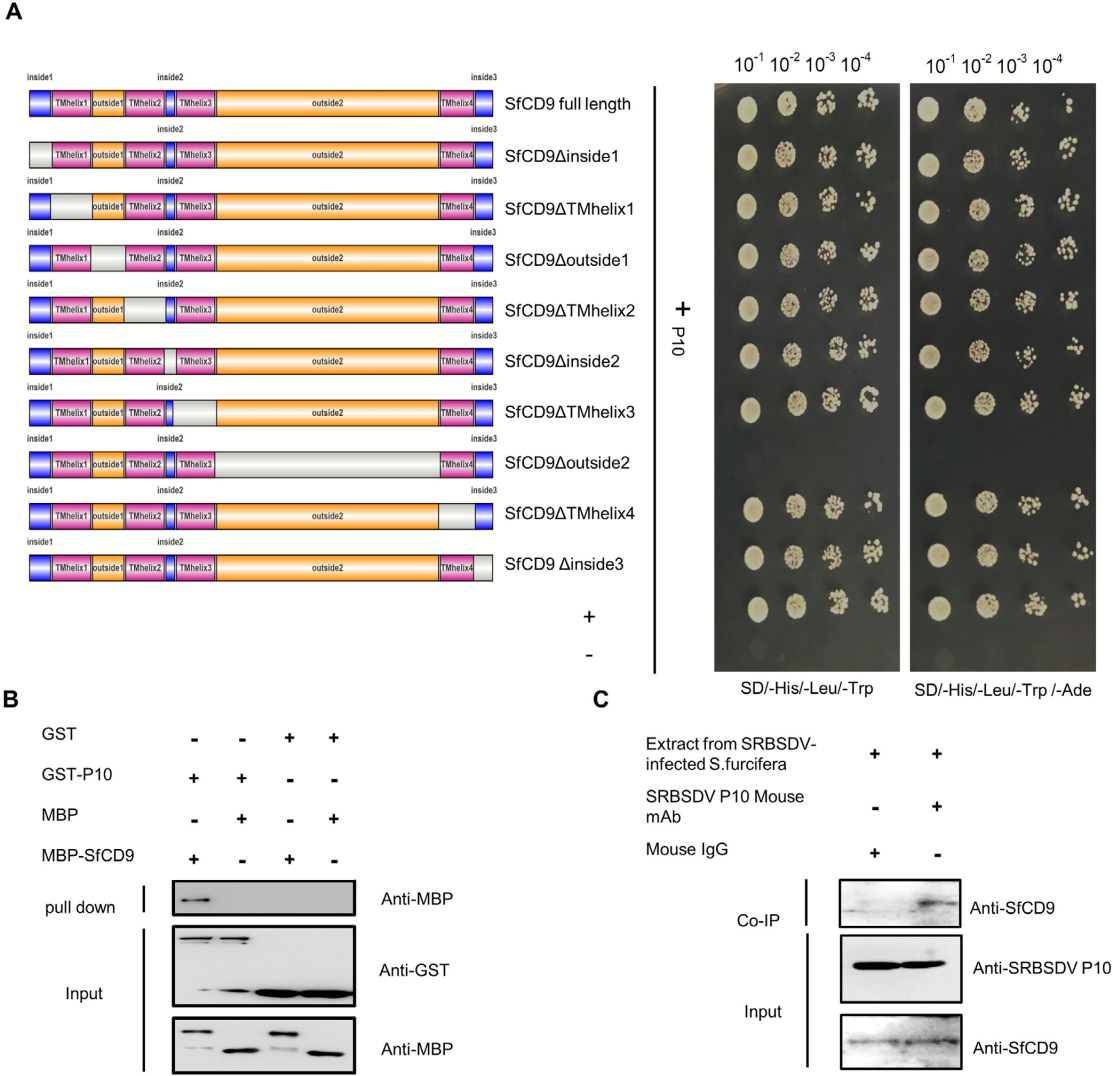
Southern rice black‐streaked dwarf virus (SRBSDV) P10 interacts with SfCD9 in vitro and in vivo. (A) Split‐ubiquitin yeast two‐hybrid assay was used to determine the interaction between SRBSDV P10 and SfCD9. Yeast cells were co‐transformed with two constructs encoding SRBSDV P10 and one of the SfCD9 truncated mutants. The transformed yeast cells were diluted from 10^−1^ to 10^−4^, and were grown for 3 days on the SD/−His/−Leu/−Trp or SD/−His/−Leu/−Trp/−Ade culture medium. The yeast cells co‐transformed with pDSL‐Δp53 and pDHB I‐large T were used as the positive control (+), while cells co‐transformed with pPR3‐N‐E and pDHB I‐large T were used as the negative control (−). (B) Glutathione S‐transferase (GST) pull‐down assay was used to determine the in vitro interaction between SRBSDV P10 and SfCD9. In this assay, the purified GST‐P10 was used to pull down maltose‐binding protein (MBP)‐SfCD9 followed by western blot assays. The resulting nitrocellulose membranes were probed with an anti‐GST or an anti‐MBP antibody. (C) Co‐immunoprecipitation analysis was used to determine the in vivo interaction between SRBSDV P10 and SfCD9. In this experiment, cell lysates from SRBSDV viruliferous *Sogatella furcifera* were immunoprecipitated with an anti‐P10 mAb or a mouse IgG followed by the detection of SfCD9 through western blot assay using an anti‐SfCD9 antibody.

To investigate the role of SfCD9 in SRBSDV invasion in *S. furcifera* , a full‐length *SfCD9* sequence was amplified through reverse transcription (RT)‐PCR and used to produce a pPR3‐N expression vector. The interaction between SRBSDV P10 and SfCD9 was further confirmed through SU‐Y2H (Figure 2A). We then aligned the SfCD9 protein amino acid (aa) sequence with CD9 sequences from *Nilaparvata lugens* , *Acyrthosiphon pisum* , *Drosophila melanogaster* , *Tribolium castaneum* , *Aedes aegypti* and *Homo sapiens* . The result showed that the SfCD9 shares the highest homology (95.11%) with the *N. lugens* CD9, and has four transmembrane helical structures that are consistent with reported tetraspanins. The tetraspanins are known to embed in cell membranes through their hydrophobic regions and to play important roles in cellular signal transduction, substance transport, cell recognition, and pathogen invasions, and their extracellular loop 2 (ECL2) has been reported as an important binding site for proteins (Rajesh et al. 2012). To further analyse this protein, we constructed multiple truncated SfCD9 mutants and analysed them through SU‐Y2H assays. The result revealed that the ECL2 region of SfCD9 did interact with SRBSDV P10 (Figure 2A).

To further confirm the interaction between SRBSDV P10 and SfCD9, we performed in vitro pull‐down and in vivo co‐immunoprecipitation (Co‐IP) assays. For the in vitro pull‐down assay, glutathione S‐transferase (GST), GST‐tagged SRBSDV P10 (GST‐P10), maltose‐binding protein (MBP) and MBP‐tagged SfCD9 (MBP‐SfCD9) were individually expressed in *Escherichia coli* cells and then purified. As shown in Figure 2B, GST‐P10 was pulled down by MBP‐SfCD9 but not by MBP, whereas GST alone could not be pulled down by MBP‐SfCD9 and GST‐P10 could not be pulled down by MBP control, confirming the direct in vitro interaction between SRBSDV P10 and SfCD9. The Co‐IP assay using total native protein extracted from viruliferous *S. furcifera* followed by western blot assay also showed that the endogenous SfCD9 was co‐immunoprecipitated by an anti‐P10 monoclonal antibody, but not by the mouse IgG as the negative control (Figure 2C), indicating that SRBSDV P10 interacts with SfCD9 in vivo. Overall, these findings indicate that SRBSDV P10 interacts with SfCD9 both in vitro and in vivo.

### 2.3 SfCD9 Colocalises With SRBSDV P10 and Relocates It From Endoplasmic Reticulum to the Cytomembrane When Co‐Expressed in Sf9 Cells

**FIGURE 3:**
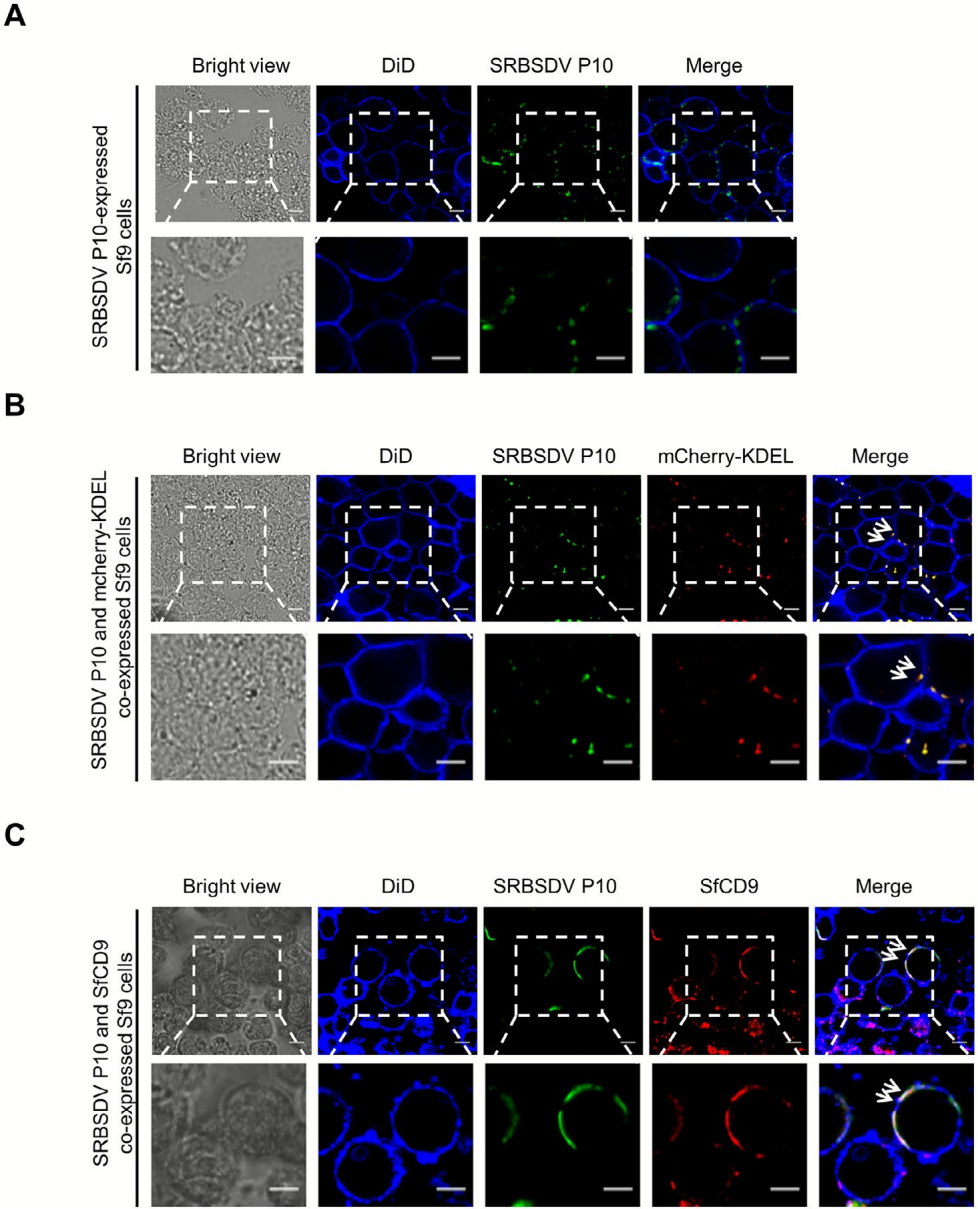
SfCD9 could alter the subcellular localisation pattern of southern rice black‐streaked dwarf virus (SRBSDV) P10 when co‐expressed in Sf9 cells. (A) SRBSDV P10 was localised in the cytoplasm of Sf9 cells. In this experiment, SRBSDV P10 was expressed in Sf9 cells and was labelled with an anti‐SRBSDV P10 mouse mAb followed by a Dylight 488‐conjugated goat anti‐mouse IgG antibody. Cell membrane of the Sf9 cells was labelled with DiD (blue). Scale bar, 10 μm. (B) SRBSDV P10 was localised in the endoplasmic reticulum (ER) of Sf9 cells. SRBSDV P10 and mCherry‐KDEL were co‐expressed in Sf9 cells. Under a confocal microscope, SRBSDV P10 was found to localise in ER labelled with the mCherry‐KDEL (red). Labelling of SRBSDV P10 and the cell membrane was the same as described in (A). Scale bar, 10 μm. (C) After co‐expressing SRBSDV P10 and SfCD9 in Sf9 cells, SRBSDV P10 was found to be localised on the cell membrane. In this experiment, SfCD9 was labelled with an anti‐SfCD9 rabbit polyclonal antibody followed by a Dylight 549‐conjugated goat anti‐rabbit IgG secondary antibody (red), and SRBSDV P10 was labelled with an anti‐SRBSDV P10 mouse mAb followed by a Dylight 488‐conjugated goat anti‐mouse IgG secondary antibody (green). Scale bar, 10 μm.

Confocal microscopy analysis showed that SRBSDV P10 was localised in the cytoplasm when expressed alone in Sf9 cells (Figure 3A). Because the P10 of rice black‐streaked dwarf virus (RBSDV), a virus of the same genus, has been shown to localise in the endoplasmic reticulum (ER) (Lu, Wang, et al. 2019), we decided to investigate whether the SRBSDV P10 also localises in the ER. In this experiment, a mCherry‐fused KDEL sequence (mCherry‐KDEL) was used to visualise the ER in cells. After co‐expressing mCherry‐KDEL and SRBSDV P10 in Sf9 cells using the baculovirus expression system, SRBSDV P10 was observed together with mCherry‐KDEL in the assayed Sf9 cells (Figure 3B), indicating that P10 is localised in the ER of Sf9 cells. To investigate the subcellular localisation pattern of SfCD9, we heterologously expressed SfCD9 in Sf9 cells followed by immunolabelling with an anti‐SfCD9 rabbit polyclonal antibody and a DyLight 549‐conjugated goat anti‐rabbit IgG secondary antibody. The cell membrane in these assayed Sf9 cells was visualised through DiD staining. Under the confocal microscope, the expressed SfCD9 protein was found on the DiD‐stained cell membrane, indicating that the SfCD9 is localised on the cell membrane.

To determine where SfCD9 and SRBSDV P10 colocalised, we expressed both proteins in Sf9 cells. Under a confocal microscope, the labelled SfCD9 and SRBSDV P10 were colocalised on the cytomembrane of Sf9 cells (Figure 3C), indicating that the subcellular localisation pattern of SRBSDV P10 can be altered from the ER to the cytomembrane by SfCD9.

### 2.4 SRBSDV P10 Colocalises With SfCD9 in *S. furcifera* Midgut Epithelial Cells

**FIGURE 4:**
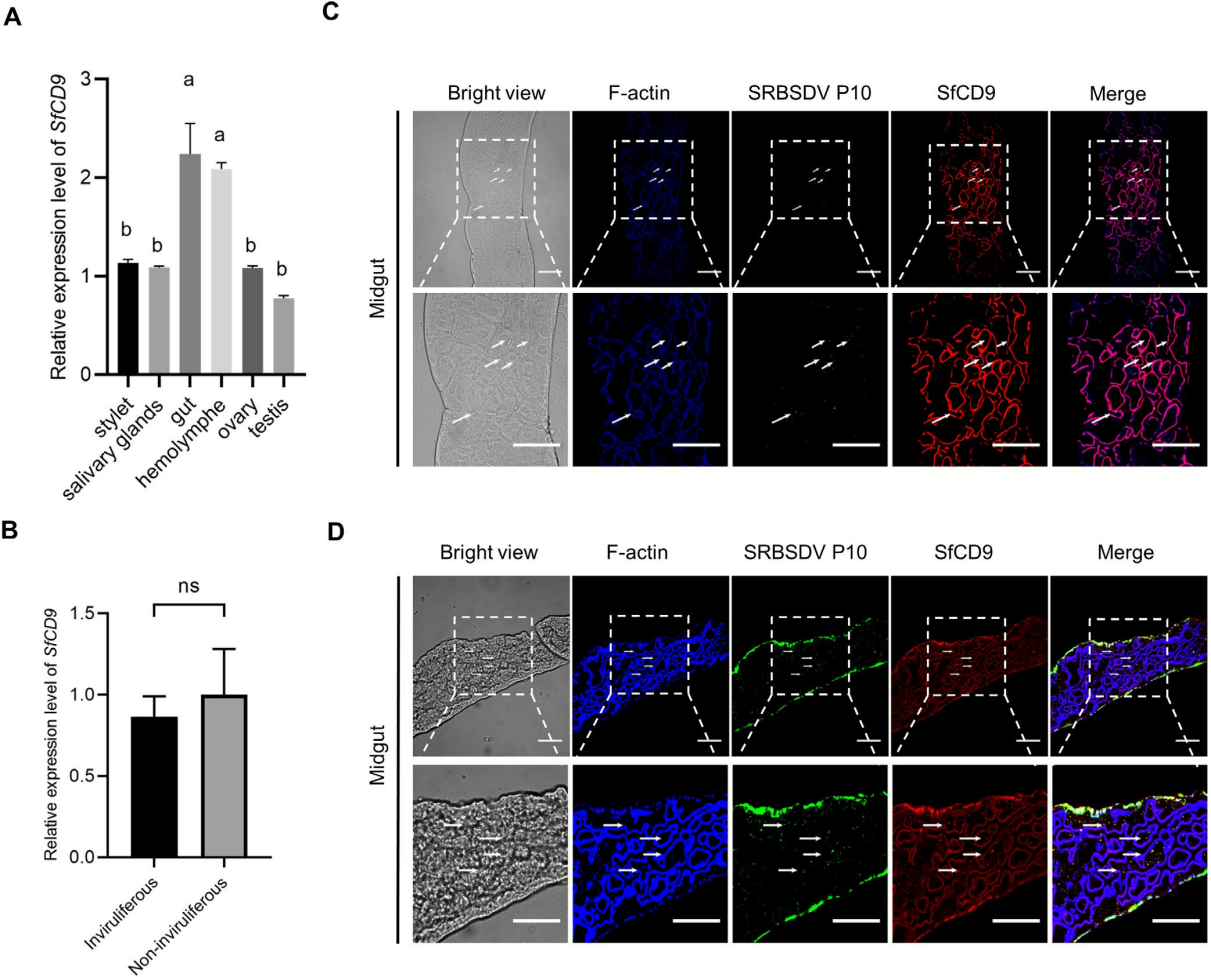
Tissue‐specific expression of SfCD9 and spatiotemporal colocalisation of southern rice black‐streaked dwarf virus (SRBSDV) P10 and SfCD9 in *Sogatella furcifera* . (A) Relative expression level of *SfCD9* in different *S. furcifera* tissues was determined through reverse transcription‐quantitative PCR (RT‐qPCR). Data are presented as means ± standard deviation (SD) (*n* = 3 biological replicates). Statistical analysis was performed using one‐way ANOVA followed by Tukey's multiple comparison test. Significant differences (*p* < 0.01) are indicated using different letters, while no significant differences are indicated by the same letter. (B) Relative expression levels of *SfCD9* in *S. furcifera* collected from virus‐infected and healthy rice was determined through RT‐qPCR. Data are presented as means ± SD (*n* = 3 biological replicates). Statistical analysis was performed using Student's *t* test. No significant differences are indicated by ‘ns’. (C) Colocalisation of SfCD9 and SRBSDV P10 at the plasma membrane of midgut epithelial cells at 2 days post‐virus feeding (dpvf). *S. furcifera* third‐instar nymphs were allowed to feed on SRBSDV‐infected rice plants for 2 days, and then midguts were collected from the assayed nymphs and analysed localisations of SfCD9 and SRBSDV P10 through immunolabelling as described for the Sf9 cells in Figure 3. Under confocal microscope, SfCD9 and SRBSDV P10 were found to colocalise on the plasma membrane of midgut epithelial cells. In this assay, the cytoskeleton of midgut epithelial cells was immunolabelled with Fluor 633‐phalloidin (blue). Scale bar, 50 μm. (D) At 6 dpvf, SfCD9 and SRBSDV P10 were found together mainly in the cytoplasm of midgut epithelial cells. In this assay, SRBSDV P10 and SfCD9 were immunolabelled as described in (C), and the cytoskeleton of midgut epithelial cells was labelled with Fluor 633‐phalloidin (blue). Scale bar, 50 μm.

Tissue‐specific expression is crucial for protein functions. To investigate the role of SfCD9 in *S. furcifera* , we analysed the expression of *SfCD9* in adult *S. furcifera* stylet, salivary gland, gut, haemolymph, testis, and ovary through reverse transcription‐quantitative PCR (RT‐qPCR). The results showed that *SfCD9* was expressed in all analysed tissues and the expression level of *SfCD9* in the gut and haemolymph was significantly higher than that in other organs (Figure 4A). Additionally, non‐viruliferous laboratory‐reared *S. furcifera* were fed on field‐collected, SRBSDV‐infected or uninfected rice plants for 14 days. Subsequently, we separately collected *S. furcifera* from SRBSDV‐infected and uninfected rice plants, and analysed the relative expression level of *SfCD9* in them using RT‐qPCR. The RT‐qPCR results revealed no significant difference in *SfCD9* mRNA expression level between viruliferous and non‐viruliferous *S. furcifera* at 14 days post‐infection (dpi) (Figure 4B).

Because the expression level of *SfCD9* in the gut and haemolymph was significantly higher than that in other organs, we decided to investigate the subcellular localisation pattern of SfCD9 in the midgut epithelial cells through immunolabelling using an anti‐SfCD9 rabbit polyclonal antibody followed by a DyLight 549‐conjugated goat anti‐rabbit IgG secondary antibody. Cytoskeleton in these cells was labelled with iFluor 633‐conjugated phalloidin. Under the confocal microscope, SfCD9 was found to be localised predominantly on the phalloidin‐labelled F‐actin cytoskeleton and a small fraction of SfCD9 was in the cytoplasm (Figure 4C).

Immunofluorescence assay was then used to explore the colocalisation of SRBSDV P10 and SfCD9 in midgut epithelial cells of SRBSDV‐infected *S. furcifera* . Third‐instar nymphs were initially fed on SRBSDV‐infected rice for 2 days, followed by transfer to healthy rice seedlings for further feeding. Confocal microscopy results showed that at 2 dpvf, SRBSDV P10 and SfCD9 were found to be predominantly colocalised on the cytomembrane of midgut epithelial cells. At 6 dpvf, however, these two proteins were colocalised predominantly in the cytoplasm of midgut epithelial cells (Figure 4C,D). Based on this finding, we hypothesised that in the early stage of invasion (i.e., 2 dpvf), SRBSDV exploits the SfCD9‐associated tetraspanin system to attach to the cytomembrane surface of the midgut epithelial cells, and then enter into the cell cytoplasm via internalisation or intracellular trafficking.

### 2.5 Silencing of *SfCD9* Expression Suppresses SRBSDV Infection

**FIGURE 5:**
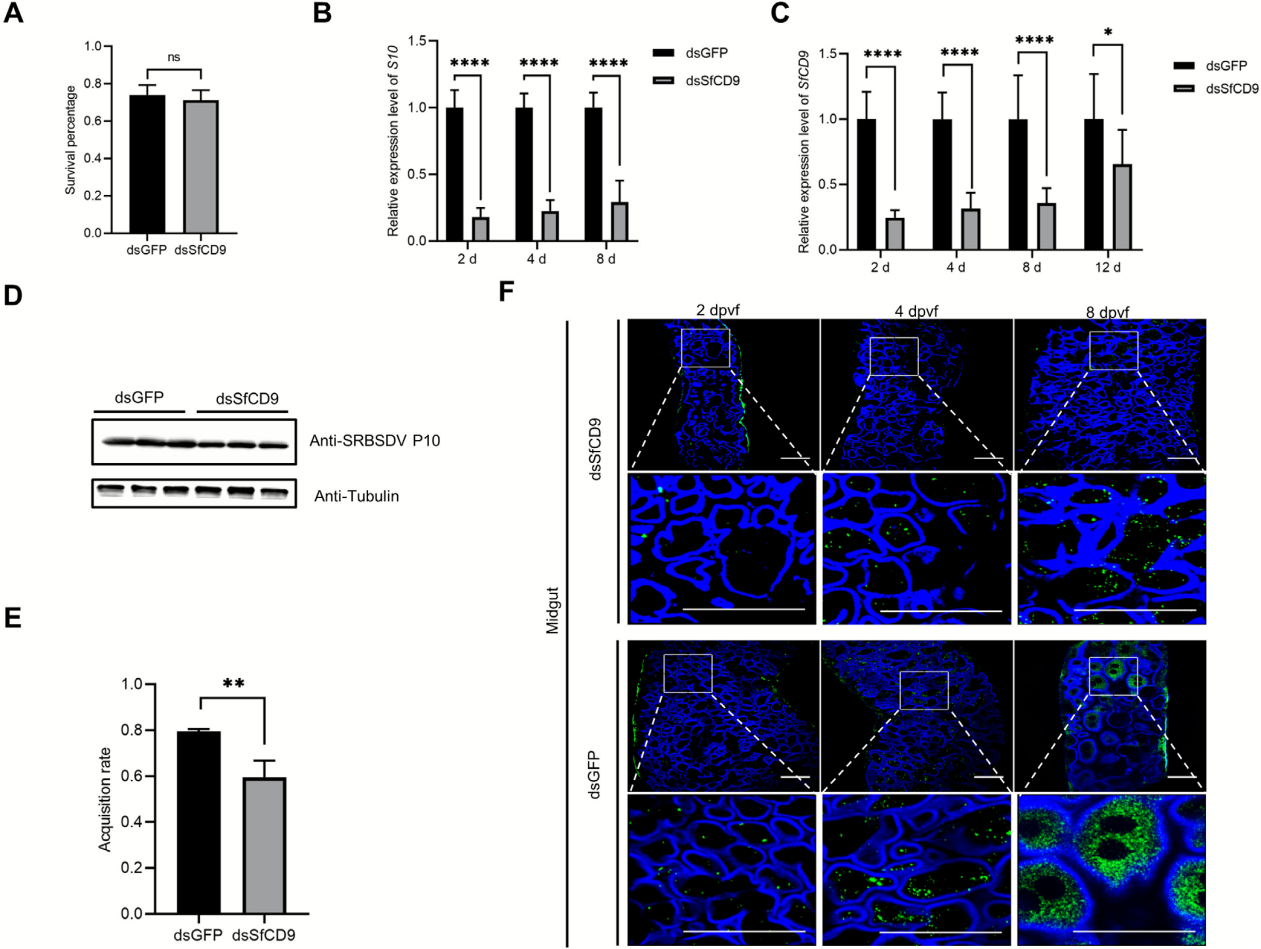
Silencing of *SfCD9* expression reduces southern rice black‐streaked dwarf virus (SRBSDV) infection in *Sogatella furcifera* . (A) The survival rates of *S. furcifera* microinjected with double‐stranded (ds)GFP or dsSfCD9 were assessed. Data are presented as the means ± SD (*n* = 3 biological replicates). No statistical difference was observed between the two treatments using Student's *t* test. (B) The expression level of *SfCD9* in *S. furcifera* microinjected with dsGFP or dsSfCD9 was analysed at 2, 4, 8 and 12 days post‐microinjection (dpm) through reverse transcription‐quantitative PCR (RT‐qPCR). The expression level of 18S rRNA was used as the internal reference. Data are presented as the means ± SD (*n* = 3 biological replicates). Statistical analysis was performed using Student's *t* test. ****p < 0.0001. (C) The expression level of SRBSDV S10 RNA in *S. furcifera* microinjected with dsGFP or dsSfCD9 was analysed at 2, 4 and 8 days post‐virus feeding (dpvf) through RT‐qPCR. Data are presented as the means ± SD (*n* = 3 biological replicates). Statistical analysis was performed using Student's *t* test. *p < 0.05, ****p < 0.0001. (D) The expression level of SRBSDV P10 in *S. furcifera* microinjected with dsGFP or dsSfCD9 was determined through western blot assays. The expression level of tubulin was used as the internal reference. (E) The virus acquisition percentage of *S. furcifera* microinjected with dsGFP or dsSfCD9 was determined. Data are presented as the means ± SD (*n* = 3 biological replicates). Statistical analysis was performed using Student's *t* test. **p < 0.01. (F) Silencing of *SfCD9* expression suppressed SRBSDV infection in vector midgut epithelial cells. SRBSDV P10 was labelled with an anti‐SRBSDV P10 mouse mAb followed by a Dylight 488‐conjugated goat anti‐mouse IgG second antibody (green), and the cytoskeleton of midgut epithelial cells was labelled with Fluor 633‐phalloidin (blue). Scale bar, 50 μm.

To further investigate the function of SfCD9 during SRBSDV invasion in *S. furcifera* , third‐instar virus‐free nymphs were microinjected with double‐stranded (ds)SfCD9 or dsGFP followed by analysing SRBSDV infection. It is noteworthy that microinjection of *S. furcifera* nymphs with dsSfCD9 did not significantly affect the survival rate of nymphs when compared to the nymphs microinjected with dsGFP (Figure 5A). The microinjected nymphs were allowed to feed on SRBSDV‐infected rice plants for 2 days and then on healthy rice seedlings. At 2, 4, and 8 dpvf, the assayed nymphs were collected and analysed for the accumulations of SRBSDV S10 RNA and P10 protein through RT‐qPCR and western blot assay, respectively. The result of RT‐qPCR showed that the expression level of *SfCD9* in the dsSfCD9‐microinjected nymphs was decreased by 20%–50% by 2–8 days post‐microinjection (dpm) (Figure 5B). By 12 dpm, the silencing level of *SfCD9* in the dsSfCD9‐microinjected insects was partially recovered, but still significantly lower than that in the dsGFP‐microinjected insects (Figure 5B). The result of RT‐qPCR also showed that the level of SRBSDV S10 RNA in the dsSfCD9‐microinjected nymphs was significantly reduced when compared to the dsGFP‐microinjected nymphs (Figure 5C). The western blot assay result agreed with the RT‐qPCR result and showed that the dsSfCD9‐microinjected insects accumulated much less SRBSDV P10 (Figure 5D). In addition, the dsSfCD9‐microinjected *S. furcifera* showed a significant reduction of SRBSDV acquisition rate (Figure 5E). Immunofluorescence assay results further revealed that silencing of *SfCD9* expression in *S. furcifera* suppressed SRBSDV accumulation in the midgut epithelial cells (Figure 5F). Therefore, we conclude that SfCD9 is a positive regulator of SRBSDV infection in *S. furcifera* .

### 2.6 Feeding *S. furcifera* With an Anti‐SfCD9 Antibody Can Prevent SRBSDV Infection

**FIGURE 6:**
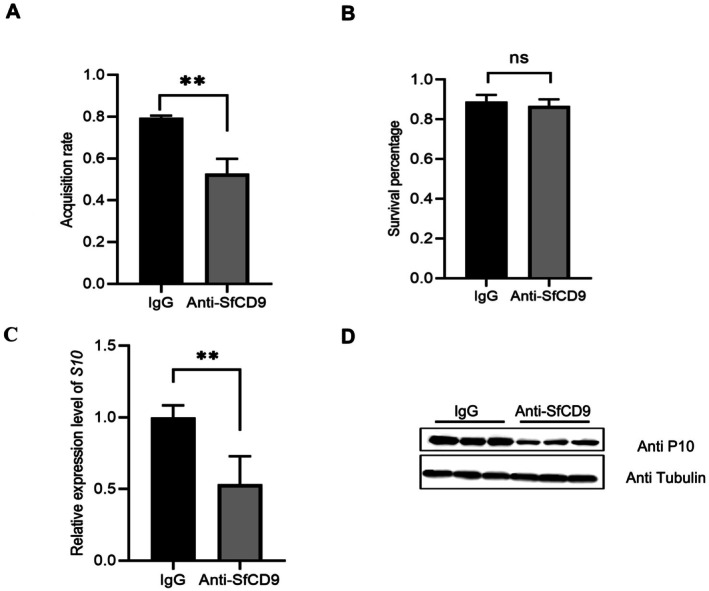
Antibody blocking of SfCD9 impaired southern rice black‐streaked dwarf virus (SRBSDV) infection in *Sogatella furcifera* . (A) Antibody feeding did not affect the survival rate of *S. furcifera* . Data are presented as the means ± SD (*n* = 3 biological replicates). No statistical difference was observed between the two treatments using the Student's *t* test. (B) The virus acquisition percentage of *S. furcifera* fed with IgG or anti‐SfCD9 antibody. *S. furcifera* was fed with an IgG or an anti‐SfCD9 antibody followed by feeding on the SRBSDV‐infected rice plants. At 12 days post‐virus feeding (dpvf), the assayed *S. furcifera* was assessed for SRBSDV acquisition rates through reverse transcription‐quantitative PCR (RT‐qPCR). Data are presented as the means ± SD (*n* = 3 biological replicates). Statistical analysis was performed using Student's *t* test. **p < 0.01. (C) The expression level of SRBSDV *S10* RNA in the IgG‐ or anti‐SfCD9 antibody‐fed *S. furcifera* was determined at 12 dpvf through RT‐qPCR. The expression level of 18S rRNA was used as the internal reference. Data are presented as the means ± SD (*n* = 3 biological replicates). Statistical analysis was performed using Student's *t* test. **p < 0.01. (D) The expression level of SRBSDV P10 in *S. furcifera* fed with IgG or anti‐SfCD9 antibody was determined through western blot assay at 8 dpvf. The expression level of tubulin was used as the control.

To elucidate whether SfCD9 can serve as a key membrane binding factor for SRBSDV entry its vector, we fed *S. furcifera* with an anti‐SfCD9 rabbit polyclonal antibody to see if this antibody could interfere with the SfCD9 and SRBSDV P10 interaction. In this antibody feeding experiment, the rabbit anti‐SfCD9 polyclonal antibody with a concentration of 1 mg/mL was mixed with the artificial diet at a 1:50 ratio and fed to *S. furcifera* for 48 h. The negative control was fed with rabbit pre‐immune serum with a concentration of 1 mg/mL. We first analysed the influence of anti‐SfCD9 antibody feeding on insect survival and found that it did not affect the survival rate of planthoppers (Figure 6A). The results of RT‐qPCR and western blot assays showed that compared with the rabbit IgG‐fed *S. furcifera* , the anti‐SfCD9 rabbit antibody‐fed *S. furcifera* showed a much lower SRBSDV acquisition rate and much lower accumulation levels of SRBSDV *P10* RNA and P10 protein in the midgut cells (Figure 6B–D), indicating that feeding the anti‐SfCD9 antibody can prevent SRBSDV infection in *S. furcifera* .

### 2.7 SRBSDV Can Enter *SfCD9* ‐Expressing Sf9 Cells

**FIGURE 7:**
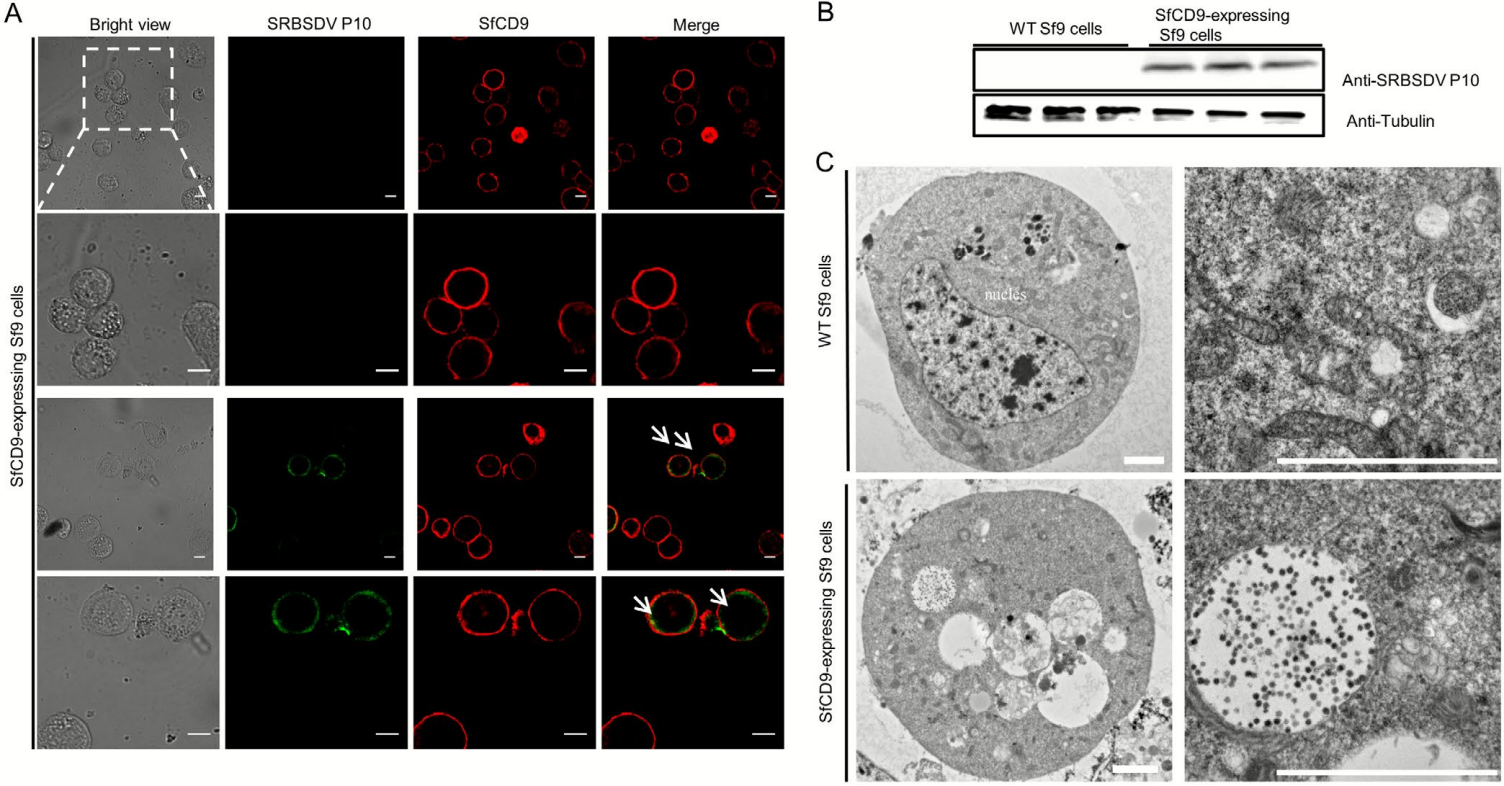
Southern rice black‐streaked dwarf virus (SRBSDV) particles can enter SfCD9‐expressing Sf9 cells. (A) Immunofluorescence assay demonstrated that SRBSDV virions can enter SfCD9‐expressing Sf9 cells. SRBSDV virions were labelled with an anti‐SRBSDV P10 mouse mAb followed by a Dylight 488‐conjugated goat anti‐mouse IgG secondary antibody (green), and SfCD9 was labelled with an anti‐SfCD9 rabbit polyclonal antibody followed by a Dylight 549‐conjugated goat anti‐rabbit IgG secondary antibody (red). The cytoskeleton of Sf9 cells was labelled with Fluor 633‐phalloidin (blue). Scale bar, 10 μm. (B) Western blot assay analysis of the accumulation level of SRBSDV P10 in wild‐type (WT) Sf9 and SfCD9‐expressing Sf9 cells after incubation with SRBSDV‐infected rice crude extract. Tubulin was used as a loading control. (C) Transmission electron microscopy showed that numerous SRBSDV virions had accumulated in *SfCD9*‐expressing Sf9 cells, but not in WT Sf9 cells. Scale bar: 2 μm.

Tetraspanins are known to possess receptor functions (Hantak et al. 2019). To explore whether SfCD9 could also act as a key membrane binding factor for SRBSDV on the cytomembrane of *S. furcifera* midgut epithelial cells, we expressed *SfCD9* in Sf9 cells. After 48 h of expression, an SRBSDV‐infected rice plant crude extract was added to the Sf9 cell medium followed by an 18 h incubation. The immunofluorescence microscope analyses showed that SfCD9 was only localised on cytomembranes of SfCD9‐expressing Sf9 cells (Figure 7A). After incubation with the SRBSDV‐infected rice plant extract, however, SfCD9 was not only localised on the cytomembrane but also in the cytoplasm of the *SfCD9*‐expressing Sf9 cells (Figure 7A). In these *SfCD9*‐expressing Sf9 cells, SRBSDV virions were predominantly discovered in the cytoplasm with a small fraction colocalised with SfCD9 on the cytomembrane (Figure 7A), suggesting that SRBSDV can use SfCD9 as the key membrane binding factor to invade the *SfCD9*‐expressing Sf9 cells. As expected, the wild‐type (WT) Sf9 cells incubated with the SRBSDV‐infected rice plant crude extract showed no green fluorescence signals (Figure 7A), indicating SRBSDV virions cannot enter WT Sf9 cells.

To further validate this finding, we analysed the accumulation of SRBSDV P10 in the *SfCD9*‐expressing or the WT Sf9 cells after incubation with the SRBSDV‐infected rice plant crude extract. Western blot assay results revealed that SRBSDV P10 was indeed accumulated in the *SfCD9*‐expressing Sf9 cells but not in the WT Sf9 cells (Figure 7B). Furthermore, we examined SRBSDV infection in the *SfCD9*‐expressing and the WT Sf9 cells through transmission electron microscopy. Under the electron microscope, numerous SRBSDV virions were observed in the *SfCD9*‐expressing Sf9 cells, but not in the WT Sf9 cells (Figure 7C), further indicating that SfCD9 is the key binding factor on the cytomembrane to facilitate the entry of SRBSDV into *S. furcifera* midgut epithelial cells.

### 2.8 SRBSDV Enters Midgut Epithelial Cells via the Clathrin‐Mediated Endocytosis

**FIGURE 8:**
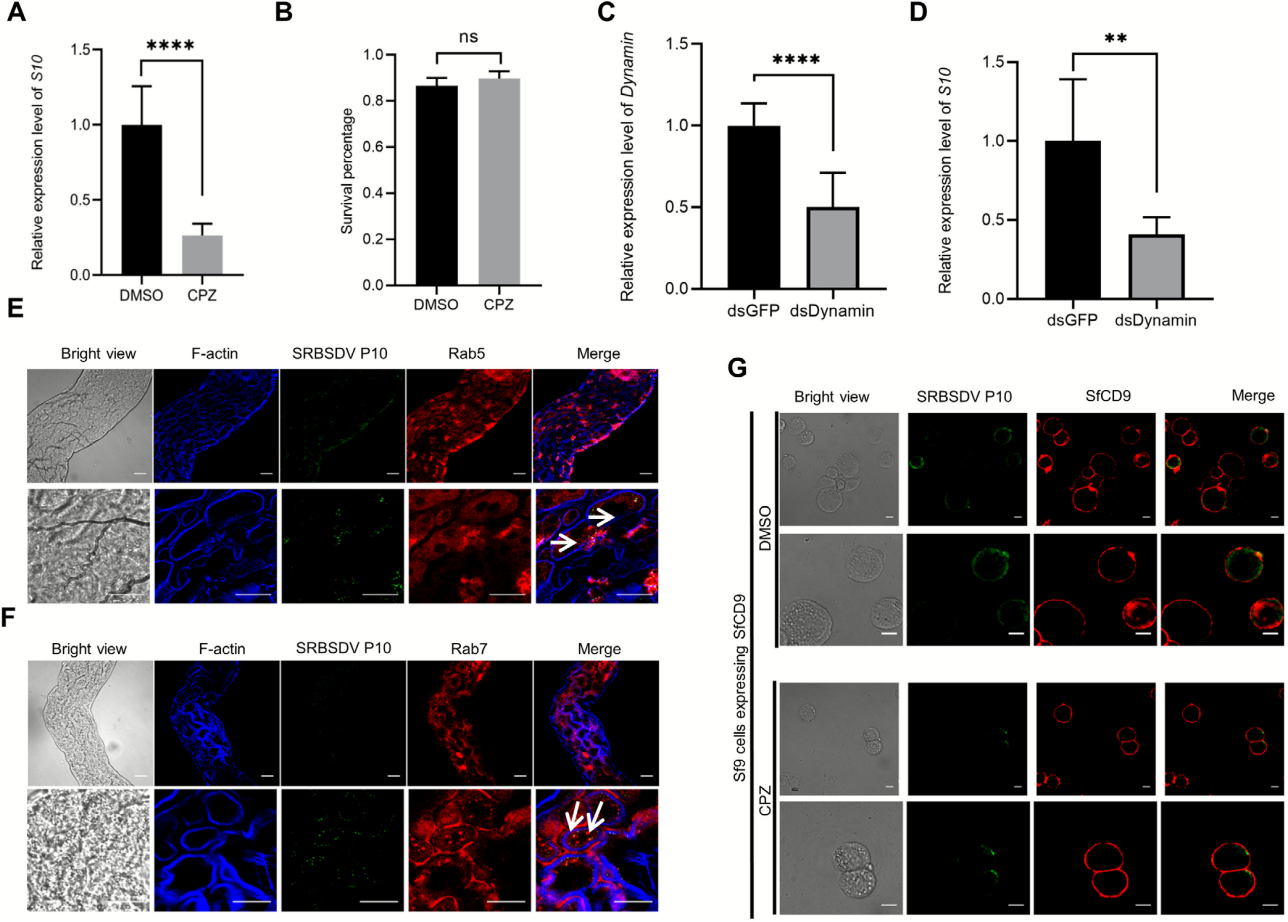
Microinjection of clathrin inhibitor chlorpromazine (CPZ) or dsDynamin significantly reduces southern rice black‐streaked dwarf virus (SRBSDV) accumulation in *Sogatella furcifera* and CPZ inhibits SRBSDV entry into SfCD9‐expressing Sf9 cells. (A) CPZ microinjection (40 μM) did not affect the survival rate of *S. furcifera* nymphs at 7 days post‐microinjection (dpm). Data are presented as the means ± SD (*n* = 3 biological replicates). No significant difference was observed between the two treatments using Student's *t* test. (B) Reverse transcription‐quantitative PCR (RT‐qPCR) analysing SRBSDV accumulation level in *S. furcifera* microinjected with CPZ after 8 days post‐virus feeding (dpvf). The expression level of 18S rRNA gene was used as the internal control. Data are the means ± SD (*n* = 3 biological replicates). Statistical analysis was performed using Student's *t* test. ****p < 0.0001. (C) The silencing efficiency of *Dynamin* gene in the dsDynamin‐microinjected or the dsGFP‐microinjected (control) *S. furcifera* was determined through RT‐qPCR. The expression level of 18S rRNA was used as an internal control. Data are the mean ± SD (*n* = 3 biological replicates). Statistical analysis was performed using Student's *t* test. ****p < 0.0001. (D) RT‐qPCR analysing SRBSDV accumulation level in dsDynamin‐microinjected *S. furcifera* . The expression level of 18S rRNA was used as the internal control. Data are presented as the means ± SD (*n* = 3 biological replicates). Statistical analysis was performed using Student's *t* test. **p < 0.01. (E) Confocal microscopy images showing the colocalisation of SRBSDV P10 protein and Rab5‐positive early endosomes at 2 days post‐infection (dpi). P10 fluorescence signals (green) was colocalised with Rab5 fluorescence signals (red). Cytoskeleton in these cells was labelled with iFluor 633‐conjugated phalloidin (blue). Scale bar, 20 μm. (F) Confocal microscopy images showing the colocalisation of SRBSDV P10 protein and Rab7‐positive late endosomes at 3 dpi. P10 fluorescence signals (green) was colocalised with Rab7 fluorescence signals (red). Cytoskeleton in these cells was labelled with iFluor 633‐conjugated phalloidin (blue). Scale bar, 20 μm. (G) CPZ treatment inhibits the entry of SRBSDV virions into SfCD9‐expressing Sf9 cells. SRBSDV virions were labelled with an anti‐SRBSDV P10 mouse mAb followed by a Dylight 488‐conjugated goat anti‐mouse IgG secondary antibody (green), and SfCD9 was labelled with an anti‐SfCD9 rabbit polyclonal antibody followed by a Dylight 549‐conjugated goat anti‐rabbit IgG secondary antibody (red). The cytoskeleton of Sf9 cells was labelled with Fluor 633‐phalloidin (blue). Scale bar, 10 μm.

Clathrin‐mediated endocytosis has been shown to play important roles in virus entry into insect cells, and the clathrin heavy chain (CHC) and light chain (CLC) as well as dynamin are critical components in clathrin‐mediated endocytosis (Pan et al. 2017). To explore whether clathrin‐mediated endocytosis could also mediate the entry of SRBSDV into *S. furcifera* midgut epithelial cells, we microinjected third‐instar *S. furcifera* nymphs with 40 μM chlorpromazine (CPZ, a clathrin‐mediated endocytosis inhibitor), dimethylsulphoxide (DMSO), dsDynamin or dsGFP. The analysis result revealed no significant difference in survival rate between the CPZ‐treated and DMSO‐treated groups (Figure 8A), demonstrating that CPZ treatment did not affect the survival rate of *S. furcifera* nymphs. Then, the microinjected nymphs were allowed to feed on SRBSDV‐infected rice plants for 2 days and then on healthy rice seedlings for 6 days. The five nymphs with the same treatment were collected as one biological sample and each RT‐qPCR had 10 biological samples. The RT‐qPCR result showed that the accumulation level of SRBSDV *P10* RNA in CPZ‐ or dsDynamin‐microinjected nymphs was significantly reduced compared to the DMSO‐ or dsGFP‐microinjected insects (control groups) (Figure 8B–D). Additionally, we used early endosome (Rab5) and late endosome (Rab7) markers to trace the trafficking of SRBSDV virions. The confocal microscopy results clearly showed that SRBSDV P10 protein was colocalised with Rab5‐positive early endosomes at 2 dpi, and colocalised with Rab7‐positive late endosomes at 3 dpi (Figure 8E,F). These results indicate that clathrin‐mediated endocytosis plays a role in the entry of SRBSDV into *S. furcifera* midgut epithelial cells.

Furthermore, CPZ‐treated Sf9 cells expressing SfCD9 revealed a weaker green fluorescence intensity compared with the control group (DMSO) (Figure 8G), indicating that the CPZ treatment inhibited the entry of SRBSDV virions into Sf9 cells. Taken together, our data suggest SRBSDV enters midgut epithelial cells through clathrin‐mediated endocytosis.

## 3 Discussion

SRBSDV is one of the most important viruses in rice‐growing areas (Matthijnssens et al. 2022). A current research on SRBSDV indicates that it can suppress the Toll immune response in *S. furcifera* by degrading MyD88 (Jia et al. 2024). Furthermore, SRBSDV uses the endoplasmic reticulum‐associated degradation (ERAD) machinery to facilitate the assembly of P7‐1 tubules, thereby promoting persistent infection and efficient transmission of SRBSDV in *S. furcifera* (Liang et al. 2022). However, to date, the molecular mechanism controlling SRBSDV entry into the midgut cells of *S. furcifera* remains largely unclear.

Through long‐term coevolution with insect vectors and host plants, viruses have evolved diverse transmission mechanisms (Whitfield et al. 2015). For example, viral capsid proteins (CPs) have been shown to play a crucial role in hemiptera‐mediated virus transmission (Wei et al. 2014). These viral CPs can directly bind to vector receptors to determine the transmission of specific viruses (Ng and Falk 2006). In 2011, the CP of lettuce infectious yellows virus (LIYV) has been shown to bind the anterior foregut of its vector *Bemisia tabaci* to promote LIYV transmission (Chen et al. 2011). Through feeding *B. tabaci* with purified recombinant tomato yellow leaf curl virus (TYLCV) CP, Wang and others found that TYLCV CP can attach to the *B. tabaci* midgut (Wang et al. 2014). In this study, we first discovered that the SRBSDV P10‐GFP fusion protein can enter midgut epithelial and haemolymph cells of *S. furcifera* , highlighting the significant role of SRBSDV P10 in viral *S. furcifera* transmission.

Through this study, we have found that SRBSDV P10 and SfCD9, a tetraspanin family member, can interact in vitro and in vivo. After co‐expressing SRBSDV P10 and SfCD9 in Sf9 cells, SfCD9 can relocate SRBSDV P10 from ER to the cytomembrane. More importantly, SfCD9 can mediate the entry of SRBSDV into the *SfCD9*‐expressing Sf9 cells, while silencing *SfCD9* expression in *S. furcifera* can prevent SRBSDV infection. Furthermore, feeding with the anti‐SfCD9 antibody can suppress SRBSDV invasion in *S. furcifera* . These findings indicate that SfCD9 plays a key role in trafficking SRBSDV into *S. furcifera* . Consequently, we propose that the interaction between SRBSDV P10 and SfCD9 on the midgut epithelial cell cytomembrane surfaces is the first step to break the intestinal barrier. We also demonstrated that SfCD9 is a crucial cytomembrane surface binding factor for SRBSDV P10 or SRBSDV virion to mediate SRBSDV virion entry into vector midgut epithelial cells via clathrin‐mediated endocytosis. To investigate whether this similar interaction occurs in other arthropod vectors, we have compared aa sequences of SfCD9 and its homologue LsCD9 from *Laodelphax striatellus* , and discovered they share a high aa identity of 98.9%. Subsequent SU‐Y2H assay results revealed that LsCD9 interacts with the related virus RBSDV P10 (Figure S1), suggesting that the tetraspanin CD9 employs a highly conserved mechanism to mediate plant virus entry into midgut epithelial cells of their insect vectors. Additionally, to investigate whether CD9 is functionally redundant with other tetraspanins, we attempted to clone other tetraspanins from *S. furcifera* , but only successfully cloned the putative *CD63* gene. Subsequent SU‐Y2H and pull‐down assay results showed that CD63 cannot interact with SRBSDV P10 (Figure S2). However, the function of CD9 and other tetraspanins in the vector transmission of other plant viruses remains to be explored in the future. Elucidating these roles will provide deeper insights into the broader role of tetraspanins in plant virus transmission and reveal new targets for intervention against vector‐borne plant viral diseases.

Several human and animal viral receptors have been reported. For example, severe acute respiratory syndrome coronavirus 2 (SARS‐CoV‐2) has been shown to employ human angiotensin‐converting enzyme 2 as the receptor to enter human cells (Conceicao et al. 2020). Tetraspanins, a family of highly conserved transmembrane proteins, serve as key facilitators of viral infection by acting as gateways for both viral entry and exit (Florin and Lang 2018). For example, human CD81, a member of the tetraspanin family, facilitates the entry of hepatitis C virus into human cells through the interaction between the viral glycoprotein and its co‐receptors (Masciopinto et al. 2001). Similarly, CD151 is crucial for the endocytosis of human papillomavirus 16 (HPV16) by interacting with the HPV16 capsid protein L2 (Scheffer et al. 2013; Wuestenhagen et al. 2016). CD81 also serves as a key receptor for porcine circovirus type 2 to invade PK‐15 cells (Li et al. 2025). Additionally, tetraspanins play a crucial role in viral transmission processes of insect vectors. For instance, tetraspanin C189 has been shown to facilitate the intercellular spread of dengue virus in mosquito vectors (Yang et al. 2015). Insect vector binding factors are also known to play decisive roles in plant virus vector transmission (Lu, Li, et al. 2019). For example, virus entry into the insect midgut and salivary gland is typically mediated by receptors that facilitate the binding of viral particles to the cell membrane surface (Czosnek et al. 2017). Rice stripe virus (RSV) virions have been reported to enter small brown planthopper (*Laodelphax striatella*) midgut epithelial cells through interaction with sugar transporter 6 and flotillin 2 (Qin et al. 2018; Wang et al. 2022). Tomato yellow leaf curl virus (TYLCV) has been shown to rely on the BtCubam receptor complex comprising BtCUBN and BtAMN to enter whitefly midgut epithelial cells via clathrin‐mediated endocytosis (Zhao et al. 2020). Another recent study has indicated that importin α2, as a plasma membrane‐associated protein, can facilitate the entry of RSV into insect salivary gland cells (Ma et al. 2021). APN‐like proteins in *B. tabaci* biotypes MEAM1 and Asia II 1 specifically interact with TYLCV and SLCMV CPs, and play a key role in determining virus transmission and vector specificity (Fan et al. 2024). In this work, we also discovered that SfCD9 is a specific membrane binding factor for SRBSDV P10 and SRBSDV virions, and promotes SRBSDV entry into *S. furcifera* .

We speculate that the high expression level of *SfCD9* in *S. furcifera* midgut is related to its key role in facilitating SRBSDV invasion in the vector, and highly expressed SfCD9 in *S. furcifera* haemolymph may help the virus evade immune elimination for virus diffusion in the vector through surrounding viral particles. This tissue‐specific distribution may reflect the virus–vector co‐evolution. Although RNAi‐mediated silencing of *SfCD9* expression or anti‐SfCD9 antibody feeding significantly suppressed virus infection in *S. furcifera* , we do not rule out the possibility that other receptors or auxiliary factors may be involved in SRBSDV invasion. Given that viral entry into cells is a highly complex process, we speculate that other tetraspanins, vitellogenin, integrins, sugar transporters, or glycoprotein receptors may also be involved in SRBSDV entry into *S. furcifera* intestinal epithelial cells (Qin et al. 2018; Ma et al. 2021; Wang et al. 2022). To investigate these hypotheses, vector factors interacting with SRBSDV P10 need to be identified through yeast two‐hybrid and Co‐IP assays, and their roles in virus invasion in *S. furcifera* will be elucidated in the future.

In conclusion, this study uncovers the critical role of SfCD9 in facilitating the entry of SRBSDV into *S. furcifera* , thereby broadening our understanding of arboviral transmission mechanisms. This conservative mechanism of tetraspanins across different species suggests that targeting tetraspanins may offer a broad application to prevent arbovirus transmission. Our findings not only expand our understanding of the transmission mechanisms of plant viruses but also provide a theoretical basis for developing novel rice virus control strategies by disrupting the virus–SfCD9 interaction.

## 4 Experimental Procedures

### 4.1 Sources of Insect, Virus and Antibodies

*Sogatella furcifera* was initially captured from a rice field, and then reared on healthy rice seedlings grown inside a growth room maintained at 26°C ± 1°C, 50%–70% relative humidity, and a photoperiod of 16 h light/8 h dark. SRBSDV‐infected rice plants were collected from a rice field and confirmed through RT‐PCR. One hundred *S. furcifera* first‐instar nymphs were fed on the SRBSDV‐infected rice plants for 2 days to establish an SRBSDV‐viruliferous *S. furcifera* population. This SRBSDV‐viruliferous population was cultured on healthy rice seedlings that were replaced once every 3 days. At 10 dpvf, the insects were sampled and tested for SRBSDV infection through dot‐ELISA (Liu et al. 2025). The viruliferous *S. furcifera* were then used to transmit SRBSDV to healthy rice seedlings as previously described (Wang et al. 2012).

The anti‐SRBSDV P10 mouse monoclonal antibody (mAb) was produced in our laboratory (Zeng et al. 2025), and the anti‐SfCD9 polyclonal antibody was also generated in our laboratory via immunising rabbits with the prokaryotically expressed SfCD9 protein. The anti‐GST mouse mAb (cat. no. AE001, Abclonal), anti‐MBP murine mAb (cat. no. AE016, Abclonal), anti‐Rab 5 rabbit pAb (cat. no. A1180, Abclonal), and anti‐Rab 7 rabbit mAb (cat. no. A12308, Abclonal) were also used in this study. DyLight 488‐conjugated goat anti‐mouse IgG (cat. no. E032210‐01, Earthox) and DyLight 549‐conjugated goat anti‐rabbit IgG (cat. no. E032320‐01, Earthox) were used in immunofluorescence assays.

### 4.2 Total RNA Isolation, cDNA Synthesis and RT‐qPCR

Total RNA was extracted from whole *S. furcifera* bodies or their individual organs (salivary glands, guts, haemolymph, ovaries and testes) using TRIzol reagent (Takara). For each total RNA extraction, the whole body of *S. furcifera* or tissues of *S. furcifera* required for the experiment were used and the contaminated genomic DNA in the samples was removed using the gDNA Remover kit included in the ReverTra Ace qPCR RT Master Mix (Toyobo). The resulting total RNA samples were reverse‐transcribed into cDNAs using the HiScript II Q Select RT SuperMix Kit as instructed (Vazyme).

RT‐qPCR was used to measure RNA expression levels on a LightCycler 480 II instrument (Roche). Each 10 μL RT‐qPCR system contained 500 ng template cDNA, 5 μL ChamQ Blue Universal SYBR qPCR Master Mix (Vazyme) and 0.20 μL of each primer (10 μM). The amplification condition was 95°C for 10 s; 45 cycles of 95°C for 10 s, 58°C for 20 s and 72°C for 20 s. All RT‐qPCR experiments were performed in technical triplicates with at least three independent biological replicates. The relative expression level of each assayed gene was expressed as the mean ± SD. Statistical differences were determined using the Student's *t* test for two treatment comparisons and one‐way ANOVA followed by Tukey's test in the Graphpad v. 8.0 software for the comparisons among multiple treatments.

### 4.3 Constructions of Expression Vectors

Full‐length SRBSDV *P10* and *SfCD9* were individually RT‐PCR‐amplified using specific primers (Table S2). The resulting products were cloned individually into the bait vector pDHBI or the prey vector pPR3‐N (Dualsystems Biotech) to produce pDHBI‐SRBSDV P10 and pPR3‐SfCD9, respectively. The full‐length *GFP*, SRBSDV *P10*, and *SfCD9* sequences were also individually cloned into the pFastBacHTB vector (Invitrogen) with a 6 × His tag. In addition, an SRBSDV *P10‐GFP* sequence was cloned into the pFastBacHTB vector.

### 4.4 Split‐Ubiquitin Yeast Two‐Hybrid Assay

The bait vector and the cDNA library were co‐transformed into yeast strain NMY51 cells followed by cultivation on the SD/−Ade−His−Leu minimal culture medium or the SD/−Ade−His−Leu−Trp culture medium for 3 days at 30°C. The positive prey vectors were individually sequenced through Sanger sequencing.

### 4.5 Prokaryotic Expression, Recombinant Protein Purification and Pull‐Down Assay

A full‐length SRBSDV *S10* sequence was cloned between the BamHI and EcoRI restriction sites in the prokaryotic expression vector pGEX‐4T‐1 to generate a GST‐tagged P10 protein (GST‐P10). The full‐length *SfCD9* sequence was cloned between the BamHI and EcoRI restriction sites in the prokaryotic expression vector pMAL‐c5e to generate an MBP‐tagged SfCD9 protein (MBP‐SfCD9). These two vectors were separately transformed into *E. coli* BL21 (DE3) cells. After 16 h induction at 16°C using a 0.5 mM isopropyl β‐D‐thiogalactoside (IPTG) (Sigma) solution, the cells were pelleted through centrifugation and then sonicated on ice for 30 min. The expressed proteins were purified using glutathione resin (GenScript) or dextrin beads (Smart‐Lifesciences) as instructed. For the GST pull‐down assay, the purified GST‐P10 fusion or GST alone was incubated with the target proteins for 1 h at 4°C with gentle rotation followed by a 2 h incubation with glutathione resin at 4°C. The mixtures were centrifuged for 5 min at 3380 *g* and the pelleted glutathione resins were washed five times in the NETN buffer containing 50 mM Tris‐HCl, 0.6% NP‐40, 150 mM NaCl, and 1 mM EDTA. The resin‐bound proteins were eluted through boiling in an SDS‐PAGE loading buffer. The resulting pull‐down products were detected through western blot assays using an anti‐MBP or an anti‐GST antibody.

### 4.6 Co‐Immunoprecipitation Assay

*Sogatella furcifera* were allowed to feed on SRBSDV‐infected rice plants for 2 days and then collected. Total protein of viruliferous *S*. *furcifera* was extracted in a cytoplasmic extraction buffer (Invent Biotechnologies). The extracted protein samples were individually incubated for 2 h with an anti‐SRBSDV P10 mouse monoclonal antibody (mAb) or a mouse IgG (Beyotime) at 4°C followed by incubation with protein A/G‐conjugated magnetic beads (Thermo Fisher Scientific) for 2 h at 4°C. The magnetic beads were captured using a magnetic holder and then washed five times in the Co‐IP buffer containing 25 mM Tris‐HCl (pH 7.5), 10% glycerol, 0.15% Nonidet P‐40, 150 mM NaCl, and a protease inhibitor cocktail. The co‐immunoprecipitated proteins were boiled for 10 min in the SDS‐PAGE loading buffer and analysed through western blot assay using an anti‐SfCD9 or an anti‐SRBSDV P10 antibody.

### 4.7 Double‐Stranded RNA (dsRNA) Synthesis and Microinjection

A DNA fragment of the *SfCD9* gene was PCR‐amplified using primers containing a T7 promoter sequence and subsequently used for in vitro dsRNA synthesis with the T7 High Yield RNA Transcription Kit (Vazyme) as instructed. Microinjection of double‐stranded *S*. *furcifera* RNA (dsSfCD9) was performed using a Nanoject III instrument (Drummond Scientific). For each microinjection, 30 nL of dsSfCD9 (1 μg dsRNA per μL) was microinjected into the *S*. *furcifera* thorax. *S*. *furcifera* microinjected with dsGFP was used as the control. The microinjected *S*. *furcifera* were reared on healthy rice seedlings for 2 days and then on SRBSDV‐infected rice plants. Silencing efficiency of *SfCD9* expression in microinjected *S*. *furcifera* was determined through RT‐qPCR.

### 4.8 Transmission Electron Microscopy

Sf9 cells were fixed overnight in 0.01 M phosphate buffer containing 2.5% glutaraldehyde at 4°C and then washed three times in phosphate buffer. The cells were post‐fixed again for 1.5 h in a 1% OsO_4_ solution. After three washes in phosphate buffer, the fixed cells were dehydrated in graded (i.e., 30%, 50%, 70%, 80%, 90%, 95% and 100%) ethanol solutions and then embedded in Spurr resin. The embedded samples were sectioned using a UC7 Ultramicrotome (Leica Microsystems), stained with uranyl acetate and alkaline lead citrate for 5–10 min. The stained sections were examined under a transmission electron microscope (H‐7820l Hitachi High‐Technologies Corporation).

### 4.9 Immunofluorescence Assay

For Sf9 cells, Sf9 cells were adhered onto cover slips and fixed for 15 min in a 4% paraformaldehyde solution at room temperature (RT). After three washes in phosphate buffer, the cells were permeabilised in phosphate buffer containing 1% Triton X‐100 for 30 min at RT. After three washes in phosphate buffer, the cells were incubated for 2 h in phosphate buffer with 5% bovine serum albumen (BSA) at RT followed by 1 h incubation in a 1:400 (vol/vol) diluted anti‐SRBSDV P10 mAb solution or a 1:400 (vol/vol) diluted anti‐SfCD9 rabbit polyclonal antibody solution at RT. The cells were then incubated for 1 h in a Dylight 488‐conjugated goat anti‐mouse IgG antibody solution or in a Dylight 549‐conjugated goat anti‐rabbit IgG antibody solution at RT. The plasma membrane of these cells was stained with DiD using a Cell Plasma Membrane Staining Kit (Beyotime) for 10 min at 37°C.

For assayed *S. furcifera* , midguts were excised and fixed for 2 h in a 4% paraformaldehyde solution at RT. The fixed midguts were permeabilised for 30 min in a 2% Triton X‐100 solution at RT. After three washes in phosphate buffer, the midguts were blocked with a 5% BSA solution for 2 h at RT and then incubated in a Dylight 488‐conjugated anti‐P10 mAb or a Dylight 549‐conjugated anti‐SfCD9 rabbit antibody for 1.5 h at RT. The resulting midgut samples were examined under a confocal microscope (Olympus).

## Author Contributions

**Shibo Gao:** methodology, investigation, validation, formal analysis, software, visualisation, writing – original draft. **Liyan Li:** methodology. **Ming Zeng:** methodology. **Li Xie:** conceptualization. **Jingjing Li:** conceptualization. **Xueping Zhou:** writing – review and editing. **Jianxiang Wu:** conceptualization, funding acquisition, writing – review and editing, project administration, resources, supervision. All authors have read and agreed to the published version of the manuscript.

## Conflicts of Interest

The authors declare no conflicts of interest.

## Supporting information


**Figure S1:** SU‐Y2H showing the interaction between RBSDV P10 and LsCD9. Yeast cells were co‐transformed with two constructs encoding RBSDV P10 and LsCD9. The transformed yeast cells were diluted from 10^−1^ to 10^−3^, and then were grown for 3 days on the SD/‐His/‐Leu/‐Trp or SD/‐His/‐Leu/‐Trp/‐Ade culture medium. The yeast cells co‐transformed with pDSL‐Δp53 and pDHB I‐large T were used as the positive control (+), while cells co‐transformed with pPR3‐N‐E and pDHB I‐large T were used as the negative control (−).

**Figure S2:** SfCD63 can not interact with SRBSDV P10.

**Table S1:** Candidate genes of *S. furcifera* screened by split‐ubiquitin yeast two‐hybrid system.
**Table S2:** Primers used in this study.

## Data Availability

The data that support the findings of this study are available from the corresponding author upon reasonable request.
