# Supplementary material for: Tetraspanin SfCD9 as a Key Membrane Binding Factor of SRBSDV P10 Facilitates Viral Entry Into Sogatella furcifera Midgut Epithelial Cells via Clathrin‐Mediated Endocytosis
Source: Mol Plant Pathol. 2025 Nov 16;26(11):e70177. doi: 10.1111/mpp.70177 (PMC12620411; doi:10.1111/mpp.70177)
Supplement: Supplementary file 1 — Figure S1: SU‐Y2H showing the interaction between RBSDV P10 and LsCD9. Yeast cells were co‐transformed with two constructs encoding RBSDV P10 and LsCD9. The transformed yeast cells were diluted from 10−1 to 10−3, and then were grown for 3 days on the SD/‐His/‐Leu/‐Trp or SD/‐His/‐Leu/‐Trp/‐Ade culture medium. The yeast cells co‐transformed with pDSL‐Δp53 and pDHB I‐large T were used as the positive control (+), while cells co‐transformed with pPR3‐N‐E and pDHB I‐large T were used as the negative control (−). [file MPP-26-e70177-s003.docx]

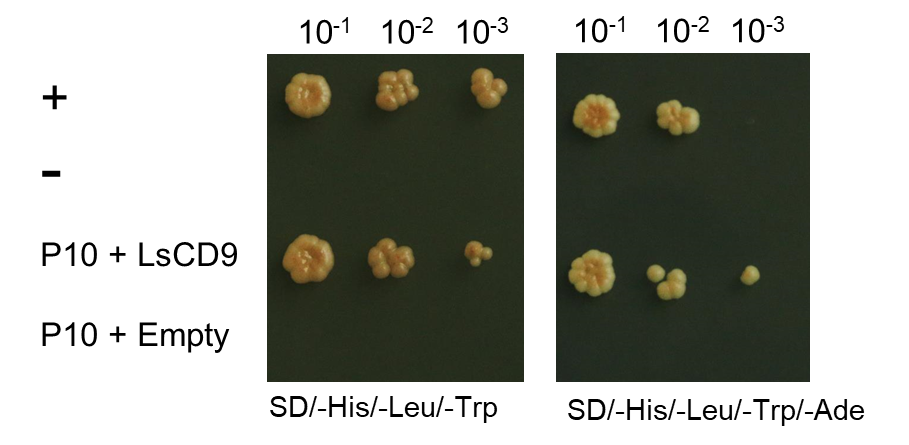


Figure S1. SU-Y2H showing the interaction between RBSDV P10 and LsCD9. Yeast cells were co-transformed with two constructs encoding RBSDV P10 and LsCD9. The transformed yeast cells were diluted from 10^-1^ to 10^-3^, and then were grown for 3 days on the SD/-His/-Leu/-Trp or SD/-His/-Leu/-Trp/-Ade culture medium. The yeast cells co-transformed with pDSL-Δp53 and pDHB Ⅰ-large T were used as the positive control (**+**), while cells co-transformed with pPR3-N-E and pDHB Ⅰ-large T were used as the negative control (-).
