# Supplementary material for: Tetraspanin SfCD9 as a Key Membrane Binding Factor of SRBSDV P10 Facilitates Viral Entry Into Sogatella furcifera Midgut Epithelial Cells via Clathrin‐Mediated Endocytosis
Source: Mol Plant Pathol. 2025 Nov 16;26(11):e70177. doi: 10.1111/mpp.70177 (PMC12620411; doi:10.1111/mpp.70177)
Supplement: Supplementary file 2 — Figure S2: SfCD63 can not interact with SRBSDV P10. [file MPP-26-e70177-s001.docx]

**
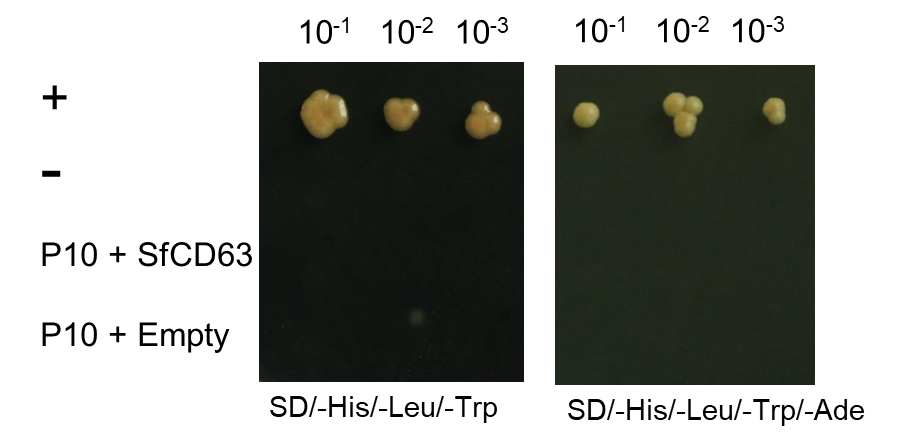
**A

B

**
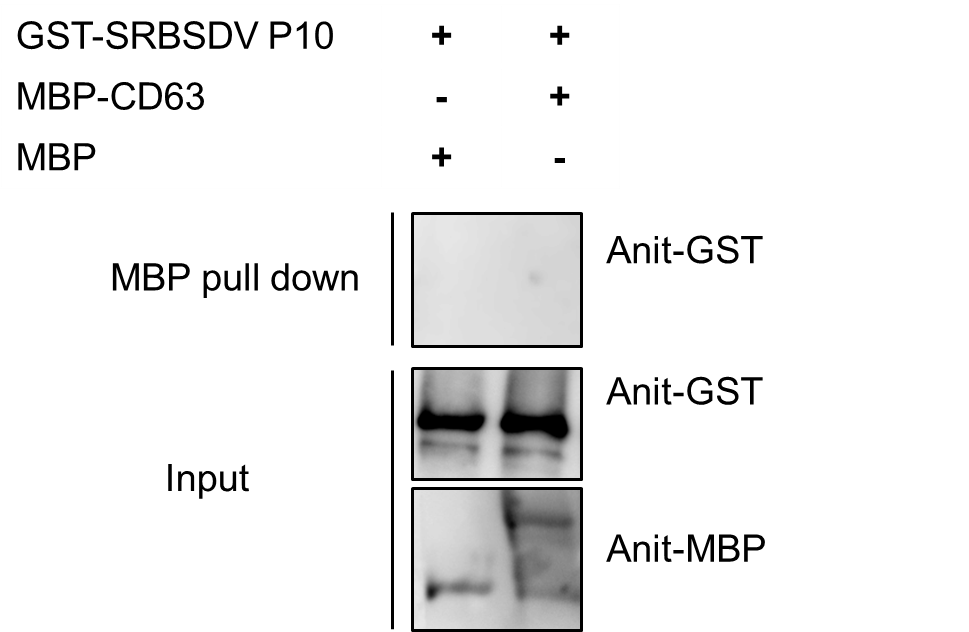
**

Figure S2. SfCD63 can not interact with SRBSDV P10.

1. SU-Y2H was used to determine the interaction between SRBSDV P10 and SfCD63. Yeast cells were co-transformed with two constructs encoding SRBSDV P10 and SfCD63. The transformed yeast cells were diluted from 10^-1^ to 10^-3^, and then were grown for 3 days on the SD/-His/-Leu/-Trp or SD/-His/-Leu/-Trp/-Ade culture medium. The yeast cells co-transformed with pDSL-Δp53 and pDHB Ⅰ-large T were used as the positive control (**+**), while cells co-transformed with pPR3-N-E and pDHB Ⅰ-large T were used as the negative control (-).
2. MBP pull-down assay was used to determine the *in vitro* interaction between SRBSDV P10 and SfCD63. In this experiment, the purified MBP-SfCD63 recombinant protein was used to pull down purified GST-P10 recombinant protein followed by Western blot detection. The resulting membranes were probed with an anti-GST or an anti-MBP antibody.
