# Supplementary material for: Tetraspanin SfCD9 as a Key Membrane Binding Factor of SRBSDV P10 Facilitates Viral Entry Into Sogatella furcifera Midgut Epithelial Cells via Clathrin‐Mediated Endocytosis
Source: Mol Plant Pathol. 2025 Nov 16;26(11):e70177. doi: 10.1111/mpp.70177 (PMC12620411; doi:10.1111/mpp.70177)
Supplement: Supplementary file 3 — Table S1: Candidate genes of S. furcifera screened by split‐ubiquitin yeast two‐hybrid system. Table S2: Primers used in this study. [file MPP-26-e70177-s002.docx]

| **Table S1 Candidate genes of *S. furcifera* screened by split-ubiquitin yeast two-hybrid system** | | | | |
| --- | --- | --- | --- | --- |
| **No.** | **Accession number** | **Protein name** | **Protein function** | **Frequency** |
| 1 | GFWP01004574.1 | Small ribosomal subunit protein RACK1 | Involved in the recruitment, assembly and/or regulation of a variety of signaling molecules. | 3 |
| 2 | GFWP01023475.1 | Vitellogenin-1 | Precursor of the egg-yolk proteins that are sources of nutrients during embryonic development. | 2 |
| 3 | GFWP01034314.1 | AP complex subunit beta | Adaptins are components of the adaptor complexes which link clathrin to receptors in coated vesicles. | 7 |
| 4 | GFWP01027532.1 | Small ribosomal subunit protein S28 | Component of the small ribosomal subunit. | 7 |
| 5 | GFWP01001852.1 | Stress induced phosphoprotein 1 | Acts as a co-chaperone for HSP90AA1 | 1 |
| 6 | GFWP01016975.1 | Nucleotide-sugar transporter | Antiporter that transports nucleotide sugars across the endoplasmic reticulum membrane in exchange for either their cognate nucleoside monophosphate or another nucleotide sugar | 4 |
| 7 | GFWP01022136.1 | Soma ferritin | Stores iron in a soluble, non-toxic, readily available form. Important for iron homeostasis | 1 |
| 8 | GFWP01017864.1 | Uncharacterized protein | Unkown | 1 |
| 9 | GFWP01023031.1 | Metallothionein | The metallothioneins are involved in the cellular sequestration of toxic metal ions. | 2 |
| 10 | GFWP01021795.1 | Transcription factor BTF3 | When associated with NACA, prevents inappropriate targeting of non-secretory polypeptides to the endoplasmic reticulum (ER). | 6 |
| 11 | GFWP01034821.1 | NADH dehydrogenase [ubiquinone] beta subcomplex subunit 8 | Accessory subunit of the mitochondrial membrane respiratory chain NADH dehydrogenase (Complex I), that is believed not to be involved in catalysis. | 2 |
| 12 | GFWP01026244.1 | Lysosomal aspartic protease | May degrade organelles involved in the biosynthesis and secretion of vitellogenin | 3 |
| 13 | GFWP01012744.1 | Uncharacterized serine-rich protein | Unknown | 4 |
| 14 | GFWP01008104.1 | Translocon associated protein subunit gamma | TRAP proteins are part of a complex whose function is to bind calcium to the ER membrane and thereby regulate the retention of ER resident proteins. | 6 |
| 15 | GFWP01000456.1 | Protein jagunal | Required for endoplasmic reticulum organization and proper vesicular traffic during vitellogenesis. Required for oocyte and bristle growth. | 4 |
| 16 | GFWP01003025.1 | Endochitinase | Digests chitin in the exoskeleton during the molting process. | 2 |
| 17 | GFWP01009900.1 | PFDN2 | Binds specifically to cytosolic chaperonin and transfers target proteins to it. | 8 |
| 18 | GFWP01034610.1 | Chemosensory protein | Modulates gene transcription; simultaneously generates both a specific activator and an inhibitor of gene transcription, capable of modulating two distinct regulatory programs during neural development. Has a role in olfactory behavior. | 1 |
| 19 | GFWP01030064.1 | AP-3 complex subunit mu | Part of the AP-3 complex, an adaptor-related complex which seems to be clathrin-associated. | 7 |
| 20 | GFWP0109813.1 | CD9 | Structural component of specialized membrane microdomains known as tetraspanin-enriched microdomains (TERMs), which act as platforms for receptor clustering and signaling. | 3 |
| 21 | GFWP01009464.1 | Integrin alpha-PS1 | Integrin alpha-PS1/beta-PS is a receptor for laminin. | 1 |
|  |  |  |  |  |

| **Table S2 Primers used in this study** | |
| --- | --- |
| **Primer Names** | **Primer Sequences (5’-3’)** |
| SRBSDV-detection-F | CGCGTCATCTCAAACTACAG |
| SRBSDV-detection-R | TTTGTCAGCATCTAAAGCGC |
| *SfCD9*-Full-F | ATGGGTCTCTCGGGATGTTATGC |
| *SfCD9*-Full-R | TAGGCCTTGTAGCGATTGCGTT |
| SRBSDV *S10*-Full-F | ATGGCTGACATAAGACTTGACAT |
| SRBSDV *S10*-Full-R | TCATCTGGTGACTTTATTTAACAC |
| pDHBⅠ-SRBSDV P10-F | AACGCGGCCATTACGGCCATGGCTGACATAAGACTTG |
| pDHBⅠ-SRBSDV P10-R | CCCCGACATGGCCGAGGCGGCCAATCTGGTGACTTTAT |
| pPR3-N-SfCD9-F | AGAGTGGCCATTACGGCCATGGGTCTCTCGGGATGTTA |
| pPR3-N-SfCD9-R | CGAGAGGCCGAGGCGGCCGGGCCTTGTAGCGATTGCG |
| pMAL-c5e-SfCD9-F | CGATATCGTCGACGGATCCATGGGTCTCTCGGGATGTTAT |
| pMAL-c5e-SfCD9-R | TAATTACCTGCAGGGAATTCCTAGGCCTTGTAGCGATTGC |
| qPCR-SRBSDV *S10*-F | TGTCGTGAAGTTCCTGCTCAA |
| qPCR-SRBSDV *S10*-F | GGTCGTAACCGCCATAGTGT |
| qPCR-*SfCD9*-F | CCGTGCATCAGGAGTATGGA |
| qPCR-*SfCD9*-R | GGAGAGGGGGATCTTGTAGGT |
| qPCR-*Sf18S*-F | GTCGTAACAAGGTTTCCGTAGG |
| qPCR-*Sf18S*-R | CATTCTCAATCAGCTTTCTTCA |
| qPCR-*Sfdynamin*-F | GTCAACCGCAGTCAGAAGGA |
| qPCR-*Sfdynamin* -R | TTGCTGGTTGAGTACACGCT |
| pFastBac-SfCD9-F | TTCAGGGCGCCATGGATCCGATGGGTCTCTCGGGATGTTA |
| pFastBac-SfCD9-R | TCGACGTAGGCCTTTGAATTCGGCCTTGTAGCGATTGCGT |
| pFastBac-P10-F | TTTCAGGGCGCCATGGATATGGCTGACATAAGACTTGACA |
| pFastBac-P10-R | CGTAGGCCTTTGAATTCTCATCTGGTGACTTTATTTAACAC |
| pFastBac-P10-GFP-F | TCAAGGCGCCATGGGATCCATGGCTGACATAAGACTTGA |
| pFastBac-P10-GFP-R | CCATGAATTCTCTGGTGACTTTATTTAACACAATCTTTTG |

# Supporting Information

Figure S1. SU-Y2H showing the interaction between RBSDV P10 and LsCD9. Yeast cells were co-transformed with two constructs encoding RBSDV P10 and LsCD9. The transformed yeast cells were diluted from 10^-1^ to 10^-3^, and then were grown for 3 days on the SD/-His/-Leu/-Trp or SD/-His/-Leu/-Trp/-Ade culture medium. The yeast cells co-transformed with pDSL-Δp53 and pDHB Ⅰ-large T were used as the positive control (**+**), while cells co-transformed with pPR3-N-E and pDHB Ⅰ-large T were used as the negative control (-).

Figure S2. SfCD63 can not interact with SRBSDV P10.
